# Impact of COVID-19-related care disruptions on blood pressure management and control in community health centers

**DOI:** 10.1186/s12889-022-14763-9

**Published:** 2022-12-08

**Authors:** Margaret Meador, Fátima Coronado, Debosree Roy, R. Curtis Bay, Joy H. Lewis, Jessica Chen, Rachel Cheung, Christopher Utman, Judith A. Hannan

**Affiliations:** 1 Director of Clinical Integration and Education, National Association of Community Health Centers, Bethesda, MD USA; 2 Associate Director for Science, Division for Heart Disease and Stroke Prevention, Centers for Disease Control and Prevention, Atlanta, GA USA; 3 Post-doctoral Research Scholar, Department of Public Health, A.T. Still University-School of Osteopathic Medicine in Arizona, Mesa, AZ USA; 4 Professor, Biostatistics, Department of Interdisciplinary Health Sciences, Arizona School of Health Sciences, A.T. Still University, Mesa, AZ USA; 5 Professor of Medicine and Public Health; Chair, Department of Public Health, A.T. Still University-School of Osteopathic Medicine in Arizona, Mesa, AZ USA; 6 Quality Improvement Program Manager, The Health Federation of Philadelphia, Philadelphia, PA USA; 7 Manager of Quality, Esperanza Health Centers, Chicago, IL USA; 8 Assistant Director, Data Analytics, HealthEfficient, Albany, NY USA; 9 Million Hearts® Senior Advisor, Division for Heart Disease and Stroke Prevention, Centers for Disease Control and Prevention, Atlanta, GA USA

**Keywords:** Hypertension, Self-measured blood pressure (SMBP) monitoring, Community health centers, Telehealth, COVID-19, Clinical quality measures

## Abstract

**Background:**

Uncontrolled hypertension is a leading risk factor for cardiovascular disease. To ensure continuity of care, community health centers (CHCs) nationwide implemented virtual care (telehealth) during the pandemic. CHCs use the Centers for Medicare & Medicaid Services (CMS) 165v8 Controlling High Blood Pressure measure to report blood pressure (BP) control performance. CMS 165v8 specifications state that if no BP is documented during the measurement period, the patient’s BP is assumed uncontrolled.

**Methods:**

To examine trends in BP documentation and control rates in CHCs as telehealth use increased during the pandemic compared with pre-pandemic period, we assessed documentation of BP measurement and BP control rates from December 2019 - October 2021 among persons ages 18-85 with a diagnosis of hypertension who had an in-person or telehealth encounter in 11 CHCs. Rates were compared between CHCs that did and did not implement self-measured BP monitoring (SMBP).

**Results:**

The percent of patients with hypertension with no documented BP measurement was 0.5% in December 2019 and increased to 15.2% (overall), 25.6% (non-SMBP CHCs), and 11.2% (SMBP CHCs) by October 2021. BP control using CMS 165v8 was 63.5% in December 2019 and decreased to 54.9% (overall), 49.1% (non-SMBP), and 57.2% (SMBP) by October 2021. When assessing BP control only in patients with documented BP measurements, CHCs largely maintained BP control rates (63.8% in December 2019; 64.8% (overall), 66.0% (non-SMBP), and 64.4% (SMBP) by October 2021).

**Conclusions:**

The transition away from in-person to telehealth visits during the pandemic likely increased the number of patients with hypertension lacking a documented BP measurement, subsequently negatively impacting BP control using CMS 165v8. There is an urgent need to enhance the flexibility of virtual care, improve EHR data capture capabilities for patient-generated data, and implement expanded policy and systems-level changes for SMBP, an evidence-based strategy that can build patient trust, increase healthcare engagement, and improve hypertension outcomes.

## Introduction

Hypertension affects nearly 1 out of 2 adults in the United States (US) and is a key risk factor for cardiovascular disease [1]. In 2020, cardiovascular diseases contributed to > 931,000 deaths in the US [2]. Uncontrolled hypertension is associated with increased risk of cardiovascular disease (CVD), a predictor of adverse outcomes from COVID-19 illness [3]. Health disparities in hypertension prevalence and management are well documented and have been further exposed and exacerbated by COVID-19 [4, 5]. Furthermore, delayed medical care has been widely reported during the pandemic [6] and care avoidance was found to be more prevalent among some of the primary populations served by community health centers (CHCs) including low-resourced communities, communities of color, and persons with underlying medical conditions such as hypertension [7].

In response to the COVID-19 pandemic, CHCs adapted by offering virtual care (telehealth) to provide continuity of care to their patient population while ensuring the health and safety of their staff and patients. Nationally, 95% of the > 1,300 CHCs funded by the Health Resources and Services Administration (HRSA) reported using telehealth in 2020 compared to 43% that were telehealth capable in 2019 [8]. Maintaining telehealth was critical to providing access to care during the pandemic. However, the lack of face-to-face encounters made obtaining and documenting blood pressure (BP) measurements necessary for hypertension screening and management challenging. Even with greater use of out-of-office self-measured blood pressure monitoring (SMBP) before the pandemic, data indicate SMBP measurements are seldom recorded in reportable fields in electronic health records (EHRs), and concerns exist about patient measurements and accurate reporting [9–11].

CHCs report information on their BP control efforts to HRSA via the Uniform Data System (UDS). UDS is a reporting system that provides standardized criteria to measure the performance and operations of CHCs delivering healthcare services to underserved communities and vulnerable populations. The system allows comparisons of annual health center and state-level hypertension data to national benchmarks [11, 12]. UDS uses electronically-specified clinical quality measures (eCQMs) from the Centers for Medicare & Medicaid Services (CMS) including CMS 165v8, *Controlling High Blood Pressure*.^1^

CMS 165v8 measures the percentage of patients aged 18–85 years who had a diagnosis of hypertension overlapping the measurement period and whose most recent BP was adequately controlled (< 140/90 mmHg). The BP reading taken at the most recent encounter of any type is included, as long as the result is during the measurement period. If multiple BP measurements are taken on the same day, the lowest systolic and diastolic values are used. CMS 165v8 specifications state that if no BP measurement is documented during the measurement period, the patient’s BP is assumed “uncontrolled”. The measure specifications require patients with hypertension to be included in the measure denominator from the day they are diagnosed, with only one eligible encounter needed for inclusion [13].

In summer 2021, nearly all 23 CHCs participating in the Million Hearts^®^ Preventing Heart Attacks and Strokes in Primary Care project led by the National Association of Community Health Centers (NACHC) noticed a sustained decrease in their BP control rate using the CMS 165v8 eCQM. However, the observed decrease was not consistent with the BP measurements of a cohort of patients with uncontrolled hypertension followed by the same CHCs. Routine quality improvement chart review found that among this cohort the average systolic BP improved from 150mmHg to 145mmHg from July 2020 – June 2021. This routine quality improvement process did not identify an increase in BP levels among those patients with hypertension who received care at the health centers, but instead identified some improvement in blood pressure. The discrepancy between the chart review and the UDS measure led to the desire to explore this phenomenon further.

## Methods

### Aim

Our study aim was to examine trends in BP documentation and control rates in CHCs as telehealth use increased during the pandemic compared with pre-pandemic period.

### Setting

A convenience sample of 11 CHCs participating in the NACHC Million Hearts^®^ project was identified in the following areas: Pennsylvania (4), New York (3), District of Columbia (DC) (2), Connecticut (1), and Illinois (1). The 11 CHCs were part of three health center controlled networks (HCCNs). Hypertension management was specifically identified as a quality improvement priority in all CHCs’ strategic plans. Even before the pandemic, these CHCs had leadership support, clinical champions, and ongoing interventions aimed at improving BP control in their practice. Additionally, five of the six CHCs from New York, DC, and Connecticut (in the same HCCN) had implemented SMBP before March 2020, including configuring structured fields in their EHRs to capture SMBP measurements in a reportable format. CHCs in Pennsylvania and Illinois had not implemented SMBP before January 2021.

As of December 31, 2020, the total population served by the 11 CHCs was 258,022 (range from 3,270 patients [DC] to 90,521 patients [NY]); in most CHCs, the patient population included > 50% non-Hispanic Black (6) or > 50% Hispanic (2) persons. Most patients (64.4%) were adults aged 18–64 years. The total number of patients aged 18–85 years with a diagnosis of hypertension in these CHCs, using International Classification of Disease (ICD) 10th Edition – Clinical Modification (CM) code I10, was 59,701.

### Study design

We conducted a data review of patients aged 18–85 years with a diagnosis of hypertension who had an encounter (in-person or via telehealth) in the previous 12 months. EHR data were abstracted using various population health management platforms (e.g., i2i Population Health, BridgeIT) and aggregated by each HCCN, yielding three data sets. Data were assessed for BP control status based on the CMS 165v8 eCQM specifications. CMS 165v8 measures the percentage of patients aged 18–85 years who had a diagnosis of hypertension overlapping the measurement period (past 12 months) and whose most recent BP was adequately controlled (< 140/90 mmHg). Per the measure specifications, BP was considered uncontrolled if the patient had a BP measurement of ≥ 140 mmHg systolic or ≥ 90 mmHg diastolic or had no documented BP measurement in the measurement period.

We used a rolling 12-month assessment to compare BP control rates from December 2019 to October 2021. This timeframe allowed us to compare trends in these metrics in the pre-pandemic (with lower telehealth use) with the pandemic period (with greater telehealth use). Because CMS 165v8 states that if no BP measurement is documented during the measurement period the patient’s BP is assumed uncontrolled, we also assessed BP control based only on documented BP measurements during this timeframe and examined the proportion of patients without a documented BP measurement. We included March 2021 as an inflection point when COVID-19 vaccines became publicly available.

To determine whether BP control varied across CHCs based on SMBP implementation, we used aggregated data from the three HCCN datasets to compare five CHCs that had not implemented SMBP prior to January 2021 (non-SMBP implementing group: Pennsylvania and Illinois; total 16,030 patients with hypertension) to the other six CHCs (SMBP-implementing group: New York, Connecticut, DC; total 43,671 patients with hypertension), five of which had implemented SMBP prior to January 2021.

Finally, to identify the types of visits (in-person, telehealth, or COVID testing) by patients with hypertension and to determine whether they had a documented BP measurement during each visit type, three of the 11 CHCs (New York, 2 and Illinois, 1) were asked to review an additional random sample of ≥ 15 EHRs of patients with hypertension who had ≥ 1 medical encounter during June 1-December 31, 2020. In total, 65 additional patient records were reviewed; the type of visit was cross tabulated with whether or how the patient had a BP measurement recorded (in a structured/reportable EHR field).

Aggregate data were plotted to show the linear distribution of proportion of patients per month with hypertension who met the CMS 165v8 criteria, who had any BP documentation, and who had no BP documentation over a period of time starting December 2019 and ending in October 2021. These were done separately for CHC groups who had implemented SMBP and who had had not implemented SMBP. Additionally, descriptive statistics (n, %) were used to present these data for three discrete months as well present data on encounter types (COVID 19-related, in-person, or telehealth) of patients with hypertension from three HCCNs. Data were presented in tables and graphs using MS Excel.

## Results

In December 2019, the overall percentage of patients with an in-person encounter and no documented BP measurement in the past 12 months was 0.5% (287/58,127) across all CHCs. By March 2021, the percent of patients with no documented BP measurement increased to 27.2% (15,961/58,656) across all CHCs, to 40.0% (6,559/16,404) in the non-SMBP implementing group, and to 22.3% (9,402/42,252) in the SMBP-implementing group. By October 2021, these percentages were 15.2% (9,293/61,010) (overall), 25.6% (4,360/17,003) (non-SMBP), and 11.2% (4,933/44,007) (SMBP).

Correspondingly, overall BP control using the CMS 165v8 eCQM decreased from 63.5% (36,915/58,127) in December 2019 to 44.7% (26,240/58,656) by March 2021 across all CHCs. BP control performance using CMS165v8 decreased in the non-SMBP implementing group from 60.0% (8,945/14,903) in December 2019 to 38.4% (6,300/16,404) by March 2021. In the SMBP-implementing group, CMS 165v8 BP control performance decreased from 64.7% (27,970/43,224) to 47.2% (19,940/42,252) during the same period. By October 2021, these percentages were 54.9% (33,491/61,010) (overall), 49.1% (8,342/17,003) (non-SMBP), and 57.2% (25,149/44,007) (SMBP).

**Fig. 1:**
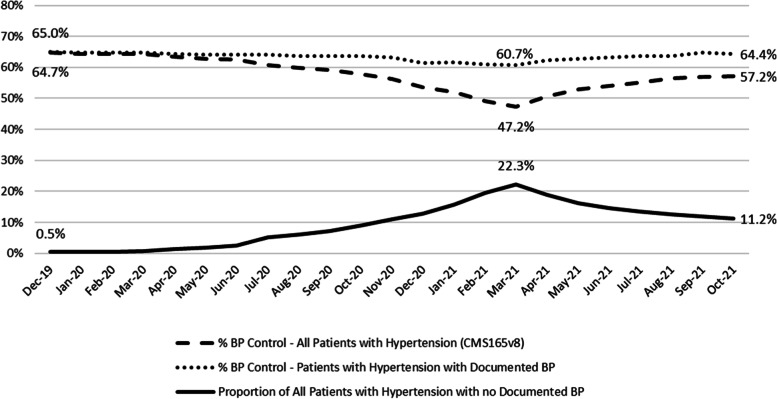
Comparison of blood pressure control among patients with hypertension using the CMS 165v8 measure specification, which deems patients as uncontrolled for hypertension if their last documented blood pressure measurement is over 12 months prior to the reporting period (even if the last recorded measurement was controlled) versus only patients with a documented blood pressure measurement in the last 12 months. The graph also shows the corresponding proportion of all patients with hypertension with no documented blood pressure measurement in the past 12 months. This comparison is among community health centers who had implemented self-measured blood pressure monitoring prior to December 2019. The reference timeframe for blood pressure control is December 2019-October 2021. The COVID-19 pandemic began impacting healthcare delivery in the United States in March 2020. In March 2021, states in the US began making COVID-19 vaccines available to all adults

**Fig. 2:**
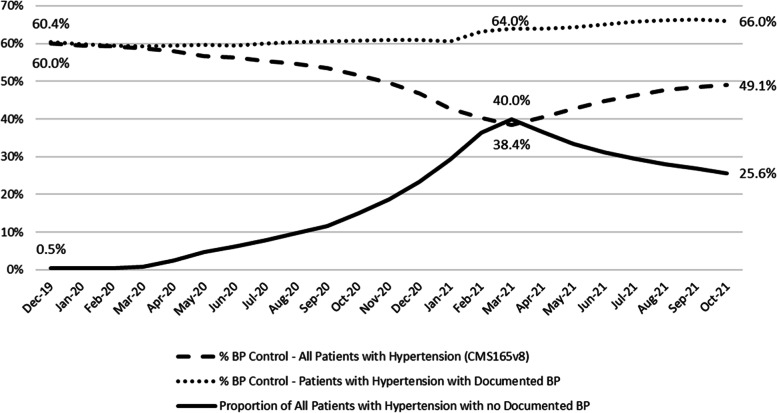
Comparison of blood pressure control among patients with hypertension using the CMS 165v8 measure specification, which deems patients as uncontrolled for hypertension if their last documented blood pressure measurement is over 12 months prior to the reporting period (even if the last recorded measurement was controlled) versus only patients with a documented blood pressure measurement in the last 12 months. The graph also shows the corresponding proportion of all patients with hypertension with no documented blood pressure measurement in the past 12 months. This comparison is among community health centers who had *not* implemented self-measured blood pressure monitoring prior to December 2019. The reference timeframe for blood pressure control is December 2019-October 2021. The COVID-19 pandemic began impacting healthcare delivery in the United States in March 2020. In March 2021, states in the US began making COVID-19 vaccines available to all adults

When assessing BP control based only on patients with a documented BP measurement in the last 12 months, data indicate that CHCs essentially maintained the proportion of patients with controlled BP. The overall BP control across all CHCs in patients with a documented BP measurement decreased from 63.8% in December 2019 (36,915/57,840) to 61.5% (26,240/42,695) by March 2021, a much smaller decrease than seen when non-documented BP patients were included. In December 2019, the BP control rate was 60.3% (8,945/14,822) in the in the non-SMBP implementing group, and 65.0% (27,970/43,018) in the SMBP implementing group. By March 2021, the BP control rate was 64.0% (6,300/9,845) for the non-SMBP implementing group, and 59.2% (19,440/32,850) for the SMBP implementing group. By October 2021, the overall rates were 64.8% (33,491/51,717); 66.0% (8,342/12,643) non-SMBP, and 64.4% (25,149/39,074) SMBP (Figs. 1 and 2 ; Table 1).

**Table 1:** Comparison of blood pressure control among patients with hypertension using the CMS 165v8 specification and among only patients with a documented blood pressure measurement in the last 12 months—Self-measured Blood Pressure-implementing (SMBP) vs. non SMBP-implementing community health centers, December 2019-October 2021

		**SMBP-Implementing CHCs**			**Non-SMBP-Implementing CHCs**			**Overall**		
		Num	Denom	%	Num	Denom	%	Num	Denom	%
December 2019	BP Control - CMS 165v8^a^	27,970	43,224	64.7%	8,945	14,903	60.0%	36,915	58,127	63.5%
	BP Control - Documented BPs^b^	27,970	43,018	65.0%	8,945	14,822	60.4%	36,915	57,840	63.8%
	No Documented BP^c^	206	43,224	0.5%	81	14,903	0.5%	287	58,127	0.5%
March 2021	BP Control - CMS 165v8	19,940	42,252	47.2%	6,300	16,404	38.4%	26,240	58,656	44.7%
	BP Control - Documented BPs	19,440	32,850	59.2%	6,300	9,845	64.0%	26,240	42,695	61.5%
	No Documented BP	9,402	42,252	22.3%	6,559	16,404	40.0%	15,961	58,656	27.2%
October 2021	BP Control - CMS 165v8	25,149	44,007	57.2%	8,342	17,003	49.1%	33,491	61,010	55.0%
	BP Control - Documented BPs	25,149	39,074	64.4%	8,342	12,643	66.0%	33,491	51,717	64.8%
	No Documented BP	4,933	44,007	11.2%	4,360	17,003	25.6%	9,293	61,010	15.2%

^a^ Numerator = Patients w/ BP control per CMS 165v8; Denominator = All patients w/ hypertension

^b^ Numerator = Patients w/ BP control; Denominator = Patients w/hypertension w/documented BP

^c^ Numerator = Patients w/o documented BP; Denominator = All patients w/ hypertension

Additional analyses of 65 EHRs from patients with hypertension from June – December 2020 revealed variations in the proportion of types of visits for these patients. In one New York CHC, telehealth visits (including both telephone and tele-video visits) comprised 13.3% of the sample, whereas in a second New York CHC telehealth visits comprised 85.0% of the sample. A third CHC in Illinois ran a large COVID-19 testing site; these visits were coded separately from traditional in-person visits. In this CHC, 33.3% of the visits were telehealth, 30.0% were in-person, and 36.7% were a COVID-19 testing encounter. Overall, 44.6% (29/65) of visits were telehealth, 38.5% (25/65) were in-person, and 16.9% (11/65) were COVID-19 testing. Among all 65 patients, BP measurements were documented in all (100%) of the in-person encounters, in approximately 10% of telehealth encounters, and in none (0%) of the COVID-19 testing encounters (Table 2).

**Table 2:** Blood pressure documentation status by encounter type for patients with hypertension, June-December 2021, n = 65

**Health Center**	**Blood pressure documented in reportable EHR field**	**Encounter Type**			**Total Number**	**Percent**
		**COVID-19 Testing**	**In-Person**	**Telehealth**		
**Total**	No	11	0	26	37	**56.9%**
	Yes - Standard Vitals Field	0	25	0	25	**38.4%**
	Yes - Telemedicine Vitals Field	0	0	3	3	**4.6%**
	**Total**	**11**	**25**	**29**	**65**	**100%**
	**Percentages **	**16.9%**	**38.5%**	**44.6%**	**100%**	
**New York 1**	No	0	0	2	2	**13.3%**
	Yes - in Standard Vitals Field	0	13	0	13	**86.7%**
	**Total**	**0**	**13**	**2**	**15**	**100%**
	**Percentages **	**0%**	**86.7%**	**13.3%**	**100%**	
**New York 2**	No	0	0	17	20	**85.0%**
	Yes - Standard Vitals Field	0	3	0	0	**15.0%**
	**Total**	**0**	**3**	**17**	**20**	**100%**
	**Percentages **	**0%**	**15.0%**	**85.0%**	**100%**	
**Illinois**	No	11	0	7	18	**60.0%**
	Yes - Standard Vitals Field	0	9	0	9	**30.0%**
	Yes - Telemedicine Vitals Field	0	0	3	3	**10.0%**
	**Total**	**11**	**9**	**10**	**30**	**100%**
	**Percentages**	**36.7%**	**30.0%**	**33.3%**	**100%**	

## Discussion

The impact of the COVID-19 pandemic on cardiovascular care has been substantial. Following the pandemic declaration, approximately 41% of US adults delayed or avoided care [6]. One of the silver linings from this pandemic has been the acceleration of innovations resulting in rapid implementation of new health care delivery models that reach patients outside of the clinic, including telehealth and increased utilization of SMBP, that would otherwise have taken years to incorporate into the workflow and improve access to care [14–16]. Nevertheless, one of the challenges of virtual care has been the decline in documented BP measurements and thus a decline in UDS reported BP control [17].

BP measurements are normally obtained by CHC clinical staff as part of routine vital signs taken during in-person encounters. In our study, in December 2019, the percent of patients with hypertension with an encounter but with no documented BP measurement in the past 12 months was minimal (0.5%). Before the pandemic, the CMS 165v8 eCQM was appropriate given the nature of the clinical encounter, endorsed timely and regular care of patients with hypertension, and was a logical gauge of BP control performance. As the pandemic progressed, we observed an inverse relationship between the increase in patients with no documented BP measurement and a decrease in BP control performance using CMS 165v8. In March 2021, reported BP control started trending upward. This may be associated with concurrent factors including the wider administration of the COVID-19 vaccine across the US, return to in-person encounters, and the wider implementation of SMBP across CHCs. By October 2021, BP control using CMS165v8 improved; however, it was still below pre-pandemic rates.

Data from a subset of patients with hypertension in three CHCs showed that BP measurements were documented on all patients with an in-person encounter, but only on 10% of those with a telehealth encounter. Although COVID-19 testing was made available at no cost at CHCs nationwide, in our study, services were limited to testing for COVID-19 and none of the patients seen through a COVID-19 testing encounter had a documented BP measurement. Although data from a national study indicate that BP control dropped from a 2019 average of 60.5 to 53.3% at the end of 2020 [16], our study suggests that telehealth and COVID-19 testing encounters likely increased the number of patients with hypertension lacking a BP measurement which might have led to lowering CHCs’ reported BP control rates. This lower performance might not be a true reflection of actual BP control in these CHCs but rather an unintended effect of the CMS 165v8 measure criteria during a period when patients increased use of virtual care options but where out-of-office measurement of blood pressure was and is still not widely practiced, accessible, or documentable in the EHR [18].

In our study, BP control based on CMS 165v8 decreased substantially in the non-SMBP implementing group relative to the SMBP-implementing group from December 2019 – March 2021. We did find that non-SMBP implementing CHCs had a higher percentage of patients with no documented BP measurement when compared to SMBP-implementing CHCs. Improvements in BP control after March 2021 were more likely a reflection of increased in-person encounters due to the availability of COVID-19 vaccines and other mitigation measures. However, over two years into the COVID-19 pandemic, lack of documented BP measurements in a substantial portion of adults with hypertension remains a challenge across CHCs, suggesting that efforts need to be made to ensure BP measurements can be documented during virtual encounters.

### Facilitators

Achieving BP control requires multiple supportive evidence-based strategies, including SMBP. Before the pandemic, 43.5% of US adults with hypertension engaged in home BP monitoring but only 6.9% shared their BP measurements with their clinical provider using remote data transmission methods [18]. Certain CHCs in this study belonged to an HCCN that had implemented SMBP among a limited number of its patients prior to March 2020. This implementation included the development of custom configurations to capture SMBP measurements in structured/reportable EHR fields, which facilitates documentation of BP measurement and corresponding hypertension management. CHCs have focused on the implementation of SMBP since 2017; with the support of HRSA’s National Hypertension Control Initiative, 500 additional CHCs nationwide received $90,000 to implement SMBP since January 2021.^2^

### Barriers

Despite the growing evidence and endorsement of SMBP’s value in diagnosing and managing hypertension [18–20], potential cost savings [21], and potential in reducing hypertension-related care and outcome disparities [22], there are well-known patient and clinician barriers for implementation. One such barrier is limited insurance coverage. As of December 2021, only 34 state Medicaid programs provided some level of coverage for automatic upper arm devices and 26 provided some level of coverage for the separate cuffs [21]. For CHCs, which receive reimbursement through Medicaid through prospective payment rates, monitors are not included, although in general, some CHCs had acquired a limited number of SMBP monitors before the pandemic through grant funding.

Although SMBP is an evidence-based strategy that, when used in conjunction with co-interventions, including medication titration, therapeutic lifestyle changes, and education and counseling, leads to clinically significant BP reduction in patients with hypertension, there is still much to be done to effect widespread implementation, particularly around data transmission [19, 23–25]. Most EHRs cannot automatically process out-of-office BP data, in part due to the lack of standards-based interfaces between home BP devices and EHRs and because of the lack of structured data fields in which to store the data. Currently, average BP is not required by the most recent version (3) of the United States Core Data for Interoperability [26], which means EHR vendors are not required to include average BP in their EHR software as a standard data field. Moreover, virtual health platforms relying on smartphones, computers, and high-speed internet may not be equally available to all patients and communities, and therefore, patients without access to or who are not literate in using the technology needed for SMBP may not benefit from this strategy.

It is also important to note that the SMBP model does not fully align with the eCQM model, which is based on assessing a single BP measurement from the most recent visit. SMBP guidelines typically ask patients to take multiple readings per day over 3–7 days and average the results, so asking the patient to take their BP again during a telehealth visit to comply with CMS 165v8 specifications may feel repetitive and not as meaningful. Further, the CMS 165v8 measure only allows for BP measurement data to be electronically transmitted from SMBP devices directly to the care team for numerator compliance; patient-reported data are not accepted [13]. This limitation precludes patients seen through telehealth encounters without the ability to show their device memory via video; it also may preclude patients without access to a Bluetooth home BP device and fast internet that enables them to share their BP measurements electronically with their health care providers. The next iteration of the UDS Controlling High Blood Pressure measure criteria to be implemented in 2023 is expected to align with the National Committee on Quality Assurance Healthcare Effectiveness Data and Information Set (HEDIS), a tool used by > 90% of US community health centers to measure performance on different dimensions of care and service, which accepts patient-reported BP measurements. Nonetheless, the issue of where to document out-of-office BP measurements in the EHR will still be a barrier, as will the incongruency of the eCQM approach of using a single BP measurement to gauge BP control vs. SMBP, which uses an average of BP readings over time to assess BP control.

### Solutions

There is an opportunity to use technology to redefine BP control by developing standards to facilitate remote data exchange between patients and health care providers and enable clinical decision making. Additionally, EHR vital signs interfaces should be improved to prompt for and allow the capture of out-of-office BP measurements and averages during a patient encounter. Collective efforts could modernize data transfer and processing, improve broadband access, expand device coverage, and increase affordability [18]. Further, an essential component to SMBP implementation involves education and training for health care providers (e.g., billing for SMBP services using current procedural terminology codes, reimbursing for SMBP training and data management), and patients (e.g., improving digital health literacy, appropriate device selection, fitting and usage, behavioral health changes). Finally, the telehealth policies that enabled broader and flexible telehealth coverage during the Public Health Emergency, especially around the originating site and audio/telephone only visits [27], could be made permanent.

### Limitations

Our study should be interpreted in the context of several limitations. First, this study was conducted during the pandemic and based on a convenience sample not generalizable to all CHCs. Our findings are mainly derived from CHCs that had existing efforts on improving BP control within their population, and therefore, should not be considered reflective of the full CHC population. Because the main purpose was to show the broad differences between groups that included people with documented BP and those that did not, we elected not to test all the differences statistically. Formal statistical testing was not conducted for two reasons. First, data were not available in a form that allowed for estimation of correlated error across multiple measurements within-patient. So, statistical tests would have violated the assumption of independence, and led to biased conclusions. Second, the sample sizes in both groups (10’s of thousands) would have yielded spuriously high significance (*p* < 0.001) for even tiny differences without conveying clinical relevance.

An additional limitation is that data were quickly extracted in the midst of the pandemic and were only available as aggregates of three HCCNs whose health centers were participating in the NACHC Million Hearts^®^ project, thus not allowing for more granular analyses. For this reason, one non-SMBP implementing CHC was analyzed as part of the SMBP implementing group, which might have underestimated the BP control rate in this group. We also did not determine the number or characteristics of patients with no encounters during the study period, nor the characteristics of patients with BP measurements and whether they differed from those who had an encounter but no documented BP. Lastly, the sample size used for the determination of encounter type was small.

## Conclusion

With disruptions in health care delivery during the COVID-19 pandemic, progress made in BP control and management—as well as care for other chronic conditions—suffered a setback. The transition away from in-person to telehealth visits during the pandemic likely increased the number of patients with hypertension lacking a documented BP measurement, subsequently negatively impacting BP control as assessed by the CMS 165v8 quality measure. Even pre-pandemic, the nation was losing ground in controlling high BP. Creative solutions are needed to address systemic causes of hypertension and engage populations and healthcare providers proactively in BP control efforts. As health care delivery in the U.S. is being transformed because of COVID-19, there is an urgent need to enhance the flexibility of virtual care, improve EHR data capture capabilities for patient-generated health data, and implement expanded policy and systems-level changes for SMBP, an evidence-based patient-centered strategy for BP control that can help build patient trust and increase healthcare engagement, as well as decrease inequities in hypertension care and outcomes.

## Data Availability

The data that support the findings of this study are available from the corresponding author upon request.

## Abbreviations

BP: Blood pressure
CDC: Centers for Disease Control and Prevention
CHCs: Community health centers
CMS: Centers for Medicare & Medicaid Services
COVID-19: Coronavirus disease 2019
CVD: Cardiovascular disease
eCQMs: Electronic clinical quality measures
EHR: Electronic health record
HCCN: Health center controlled network
HEDIS: Healthcare Effectiveness Data and Information Set
NACHC: National Association of Community Health Centers
SMBP: Self-measured blood pressure
UDS: Uniform Data System

## Footnotes

1 1Measure specifications for the CMS165v8 can be found here: Controlling High Blood Pressure (healthit.gov).

2 HRSA FY 2021 Supplemental Funding for Hypertension (NHCI-HC) can be found here: https://bphc.hrsa.gov/funding/funding-opportunities/fy-2021-supplemental-funding-hypertension-nhci-hc.
